# A survey of dystocia in the Boxer breed

**DOI:** 10.1186/1751-0147-49-8

**Published:** 2007-03-21

**Authors:** Catharina Linde Forsberg, Gunilla Persson

**Affiliations:** 1 Department of Clinical Sciences, Division of Reproduction, Faculty of Veterinary Medicine and Animal Sciences, P.O Box 7054, Swedish University of Agricultural Sciences, SE-750 07 Uppsala, Sweden; 2 Regional Animal Hospital in Helsingborg, PO Box 20097, SE-250 23 Helsingborg, Sweden

## Abstract

**Background:**

Dystocia occurs more commonly in some breeds of dogs than others. The Boxer breed is one of the highrisk breeds for whelping problems. The aim of this study was to document some reproductive parameters and the frequency of dystocia in Boxers.

**Methods:**

Two questionnaires were sent to the breeders of Boxers in Sweden during 1994 to 1997. Data from 253 whelpings and 1671 pups was received, which constitutes 56.5% of all Boxer litters registered with the Swedish Kennel Club during these years. Data was analysed using Chi-square test, and Fischer's exact test.

**Results:**

Dystocia occurred in 32% of the individual bitches, and in 27.7% of all the whelpings. Caesarian section was performed in 22.8% of all the whelpings and in 80.1% of the cases of dystocia. Medical treatment was tried in 20 cases but was successful only in 5 (25%). The dystocia was of maternal origin in 68.6% and of fetal origin in 28.6% of cases. The most common reasons for dystocia were primary uterine inertia (60%) and malpresentation of the fetus (26%). Dystocia increased with increasing age of the bitch from four years of age. Average litter size was 6.6 (± 2.2) pups born, and 5.0 (± 2.1) pups registered. Pup mortality was 24%. Stillbirths accounted for 6.1% of the pup deaths and 1% died in the neonatal period, while 15.6% of the pups were euthanised, the majority because they had disqualifying white coat colour. Cryptorchidism was observed in 9.8% of the male pups born and in 13.4% of the male pups that were registered.

**Conclusion:**

The Boxer suffers a high frequency of dystocia, mainly due to uterine inertia, but also fetal malpresentation. Breeders should be adviced to include easy whelpings in their breeding program.

## Background

Dystocia is defined as difficult birth or the inability to expel the fetus through the birth canal without assistance. Traditionally, dystocia is classified as being of either maternal or fetal origin, or a combination of both. In a study of 182 bitches of many different breeds that were brought to a veterinary hospital because of dystocia, Darvelid & Linde-Forsberg (1994)[1] found that 75.3% of the cases of dystocia had a maternal cause while 24.7% were of fetal origin. Previously Gaudet (1985) [2], in a similar survey reported that 60% of dystocias were due to maternal causes while 40% had foetal causes. The overall incidence of dystocia in the bitch is probably below 5 per cent, but it may amount to almost 100 per cent in some breeds of dogs, especially those of the achondroplastic type and those selected for large heads [1,3-7]. Gill (2002) [5], studying 15 breeds of dogs found dystocia to vary in frequency from 9.1% in Golden Retrievers to 85.7% in Pekingese, and Caesarian sections from 5.9% in Rough Collies to 60% in Pekingese. Eneroth et al. (2000) [4] reported that the frequency of Caesarian sections in Boston Terriers was 62%, and in French Bulldogs 43%. Recently, Bergström et al. (2006) [8], using data from insurance claim records from almost 200,000 insured bitches in the period 1995–2002, estimated the overall incidence of dystocia to be around 16%, and Caesarian section was performed in 63.8% of cases.

Uterine inertia is by far the most common cause of dystocia in dogs. It is classified into primary and secondary inertia. Primary complete uterine inertia is the failure of the uterus to begin labor at full term. Primary partial uterine inertia occurs when there is enough uterine activity to initiate parturition but it is insufficient to complete a normal birth of all fetuses, in the absence of an obstruction. Primary inertia can also be due to that the uterus fails to respond to the fetal signals because there are only one or two pups and thus insufficient stimulation to initiate labor (the single-pup syndrome) or because of overstretching of the myometrium by large litters, excessive fetal fluids, or oversized fetuses. Other causes of primary inertia may be an inherited predisposition, nutritional imbalance, fatty infiltration of the myometrium, age-related changes, deficiency of neuro-endocrine regulation, or systemic disease in the bitch. Secondary uterine inertia implies that some fetuses have been delivered while the remaining ones are left in utero due to exhaustion of the uterine myometrium caused by obstruction of the birth canal; this condition should be clearly distinguished from primary inertia [see [6]]. In the study by Darvelid & Linde-Forsberg (1994) [1] 72.0% of the bitches suffered from primary uterine inertia, compared to 42.1% of the bitches in the study by Gaudet (1985) [2].

The Boxer breed is known to have a high risk for dystocia [5,8]. The Swedish Boxer Club, therefore, sent a questionnaire [See Additional file 1] to all the Swedish Boxer breeders registering a litter of pups in 1994 and 1995, asking for details about the bitch's oestrous cycle, the mating and the whelping and about the development of the pups until the time of delivery to the new owners at 8 weeks of age. An unexpectedly high frequency of Caesarean sections was discovered and this prompted a follow-up survey [See Additional file 2] for the years 1996 and 1997, with more detailed questions about the parturitions and the pups. The Club then turned to the Division of Reproduction at the Swedish University of Agricultural Sciences in Uppsala for help to investigate the nature and cause of the whelping problems of the Boxer breed in order to improve their breeding program and reduce the risk for dystocia in this breed.

## Methods

### The questionnaires

The two questionnaires were sent to all the breeders that registered a litter of Boxer pups with the Swedish Kennel Club between 1994-01-01 and 1997-12-31. Breeders of litters registered in 1994 and 1995 received a version [See Additional file 1] wich contained questions about the oestrous cycle of the bitch and the mating, the course of the parturition, and about the pups during the period from birth to delivery to the new owners. Breeders that registered litters during 1996 and 1997 received another version [See Additional file 2] in which the questions were more concentrated around the whelping as such. The questionnaires were distributed to the breeders in June 1996, and in July 1998, respectively. In 22 cases the breeders were also contacted over the telephone by the authors for further clarifications.

### The oestrous cycle of the bitch

Questions about the oestrous cycles of the bitches were included in the first questionnaire and have been compiled without further analysis.

### The age of the bitch at the time of whelping

The age of the bitch at the time of whelping was calculated based on the year of registration of the bitch with the Swedish Kennel Club and the year of whelping. If the bitch's kennel prefix indicated that it was imported and, thus, registered with the Swedish Kennel Club as an adult, the year of birth was checked and used for the calculation of the bitch's age at whelping.

### Definition of dystocia

Bitches considered by their owners to be in need of veterinary treatment during whelping were, in this study, defined as suffering from dystocia.

### Duration of the whelping

In this study the duration of the whelping has been defined as the time from the birth of the first pup until the birth of the last pup.

### Classification of uterine inertia

Based on the breeders' information, bitches which had not produced any pup and for which no information was given to indicate that the first water bag had burst at the time of admission to the veterinary clinic, were classified as suffering from primary total uterine inertia. Bitches that had given birth to at least one pup before needing veterinary assistance, as well as those in which the first water bag had burst but no pup had been produced, were classified as suffering from primary incomplete uterine inertia.

### The litters

Information about the number of litters, registered pups and the sex distribution was obtained from the Kennel Club registry from the years 1994 to 1997. As the two questionnaires differed slightly (for instance regarding the sex distribution among the pups which was only included in the second questionnaire) and because not all breeders answered all the questions, the calculations have been based on those litters for which all the necessary information was available.

### Cryptorchidism

Cryptorchidism is a common defect in the Boxer breed and was therefore included in the surveys. Breeders tend to believe that cryptorchidism is more common in litters containing many male pups, and consequently we also wanted to study whether the number of pups and the sex distribution had any influence on the incidence of cryptorchidism in the litters.

### Statistical methods

Chi-square test was used to analyse relations between the discrete variables. When the values in the cells were too low for this test to be applicable, Fischer's exact test was used. A Chi-square trend analysis was used to look at the relation between a discrete variable and another discrete ordinal variable. To analyse pup death rates for parturitions of different durations we used a hypothesis test that compared two proportions. Values are presented as means ± standard deviation (S.D.) and were considered to be statistically different when P < 0.05.

## Results

Out of a total of 448 Boxer litters registered by the Kennel Club during the years of the study, 253 (56.5%) are included in the surveys.

The recorded whelpings were out of 194 individual bitches, mated to 106 different males. For 141 of the bitches there is only information about one litter each, whereas 47 bitches gave birth to two litters and six bitches to three litters each during the period of study. The study includes 1671 pups born and 1274 that were registrered with the Kennel Club.

**Figure 1:**
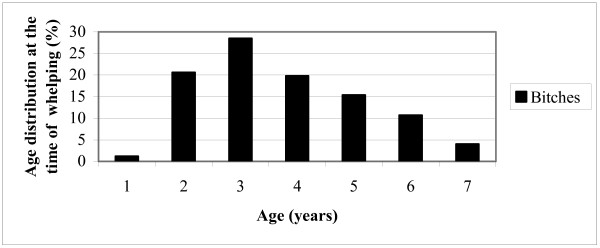
Age distribution among the bitches at the time of whelping (194 bitches; 253 whelpings).

The age distribution among the bitches at each time of giving birth is shown in Figure 1.

Most of the bitches (110 out of 118 = 93.2%) were said to exhibit regular oestrous cycles. Median interoestrous interval was 7 months, and 85% of the bitches that exhibited regular cycles had an interval of between 6 and 8 months. According to the breeders 114 out of 118 bitches (96.6%) were easy to mate. Fifteen bitches out of 105 (14.3%) were said to have failed to become pregnant at some previous mating.

In 70 out of 253 whelpings (27.7%) the bitch received veterinary treatment in connection with parturition. Altogether 62 (32.0%) of the individual bitches required veterinary assistance.

The dystocias were of maternal origin in 68.6% of the cases, and 28.6% were of fetal origin while in 2.9% of the cases it was not possible to determine whether the dystocia was maternal or fetal in origin (Table 1).

**Table 1:** Classification of dystocias (n = 70)

Cause of the dystocia	Number of whelpings	Dystocias (%)
**Maternal dystocias**	**48**	**68.6**
Primary uterine inertia	42	60.0
Incomplete uterine inertia	33	47.1
Complete uterine inertia	8	11.4
Non-classifiable primary uterine inertia	1	1.4
Obstruction of the uterine horn (the same bitch twice)	2	2.9
Uterine horn torsion	1	1.4
Placental separation/green discharge	1	1.4
Internal haemorrhage	1	1.4
Bitch accumulating too much body fluid before parturition	1	1.4
**Fetal causes**	**20**	**28.6**
Malposition	18	25.7
Fetal oversize	2	2.9
Malformation (fetal duplication)	1	1.4
**Unknown cause**	**2**	**2.9**
Obstruction of pup, for unknown reasons	2	2.9

**Figure 2:**
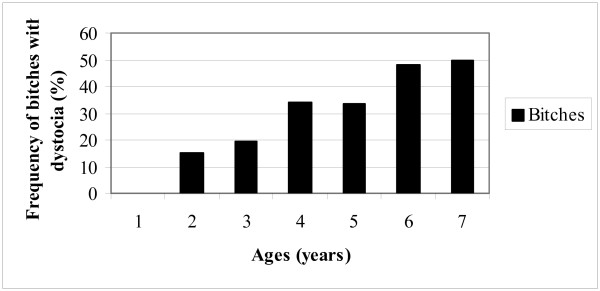
Age distribution at time of whelping for bitches needing veterinary treatment (n = 70).

**Figure 3:**
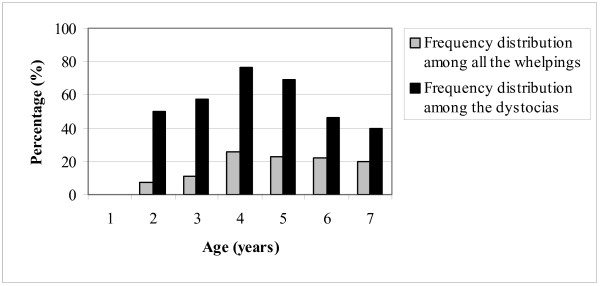
Frequency distribution of the age of the bitches at the time of whelping (n = 253) and among bitches with dystocia (n = 70).

The need for veterinary treatment increased with increasing age of the bitch (Figure 2), (chi-square trend analysis: P = 0.00007). The age distribution of the 42 cases of primary uterine inertia (16.6% of the total number of whelpings, 60.0% of the dystocias) is shown in Table 2 and Figure 3. In bitches younger than four years, 12 out of 139 (9.8%) suffered from primary uterine inertia, while among those older than four years this occurred in 30 out of 156 bitches (19.2%) (P = 0.00931).

**Table 2:** Incidence of primary uterine inertia distributed by age group (n = 42)

Age of bitch (years)	Whelpings with uterine inertia (no.)	Frequency distribution among all the whelpings (%)	Frequency distribution among the dystocias (%)
2	4	7.7	50.0
3	8	11.1	57.1
4	13	26.0	76.5
5	9	23.1	69.2
6	6	22.0	46.2
7	2	20.0	40.0

Twenty-four of the 53 bitches (45.3%) that gave birth to more than one litter of pups during the period of study received veterinary treatment during at least one of the whelpings (Table 3). For various reasons seven out of the 53 bitches (13.2%) needed veterinary treatment during more than one of the whelpings (Table 4).

**Table 3:** Need for veterinary treatment of bitches with more than one whelping during the period of study (n = 53)(means ± SD)

Order of the litter among litters included in the study	Bitches needing veterinary help/total number of bitches with more than one litter	Bitches needing veterinary help (%)	Mean age of bitch at the time of whelping (years)
First	9/53	16.9	3.2 (± 1.3)
Second	20/53	37.7	4.3 (± 1.3)
Third	3/6	50.0	5.3 (± 0.8)

**Table 4:** Causes of dystocia in bitches needing veterinary treatment during more than one of the whelpings included in this study. (n = 7)

	Whelping no. 1	Whelping no. 2	Whelping no. 3
Bitch no. 1	Haemorrhage	Primary uterine inertia	Primary uterine inertia
Bitch no. 2	Primary uterine inertia	Primary uterine inertia	
Bitch no. 3	Fetal obstruction	Primary uterine inertia	
Bitch no. 4	Oversized fetus	Primary uterine inertia	
Bitch no. 5	Fetal malposition	Primary uterine inertia	
Bitch no. 6	Fetal malposition	Fetal malposition	
Bitch no. 7	Obstructed birth canal	Obstructed birth canal	

The time from when the breeders noted the first discharge of fetal fluids and until the first pup was born was reported for 96 out of 119 whelpings (80.7%) and varied between zero min and 12 hrs (Table 5). The duration of the whelping was recorded in 168 cases (Table 6). Seventeen of the bitches for which the duration of the whelping was recorded experienced a parturition exceeding 12 hrs. Fifteen of these bitches (88.2%) received veterinary treatment. The percentage of stillborn pups in relation to the duration of the whelping could be calculated for 168 of the litters (Table 7). Forty-five of the 84 whelpings that exceeded five hrs duration lasted for more than eight hrs. The pup death rate in the whelpings lasting for more than eight hrs was, on average, 13.3%.

**Table 5:** Time between the first discharge of fetal fluids and the birth of the first pup (n= 96 whelpings)

	For all the whelpings (n = 96)	Bitches not needing veterinary treatment (n = 72)	Bitches needing veterinary treatment (n = 24)
Median time (range)	1.3 hrs (0 min – 12 hrs)	1.0 h (0 min – 8 hrs)	2.0 hrs (20 min – 12 hrs)
Mean time	1.9 hrs	1.5 hrs	3.2 hrs

**Table 6:** Duration of the whelpings from birth of the first pup (168 whelpings)(hours)

	All the whelpings (n = 168)	Whelpings without veterinary treatment (n = 129)	Whelpings with veterinary treatment (n = 39)
Median time (range)	5.0 hrs (1 h – 72 hrs)	5.0 hrs (1 h – 17 hrs)	11.0 h (2 hrs – 72 hrs)
Mean time	7.5	5.9	13.7

**Table 7:** Pup death rate in relation to the duration of the whelping from birth of the first pup in 168 litters (P < 0.0001)

Duration of the whelping (hrs)	No. of litters	Stillborn pups/total no. of pups	Stillborn pups (%)
≤ 5	84	20/481	4.2
> 5	84	67/622	10.8

In 20 out of the 70 dystocia cases (28.6%) the bitches were given medical treatment. Nine bitches were treated with both calcium and oxytocin, six were given calcium only and five were treated only with oxytocin. In fifteen of those 20 cases (75%) the bitches ended up having a Caesarian section. In four of the 70 cases of dystocia (5.7%) attempts were made to deliver the pups with the aid of uterine forceps. This treatment was successful in two cases (50%), while two of the bitches were submitted for a Caesarian section. A total of 58 litters were delivered by Caesarian section, corresponding to 22.9% of all the whelpings in the study and 80.1% of the cases of dystocia. In 58% of the cases, Caesarian sections were performed without attempting other methods for assisted delivery.

The mean litter size was 6.6 (± 2.2) pups per litter. The mean number of pups per litter at the time of registration with the Kennel Club was 5.0 (± 2.1) pups, 2.6 males and 2.4 females (sex ratio = 104). In the 116 litters for which also the sex distribution at the time of birth was reported means of 3.5 males and 3.1 bitches were born per litter (sex ratio = 106), and 2.6 males and 2.5 bitches were subsequently registered (sex ratio = 102).

Twelve of the litters in this study consisted of only one or two pups, and in six of those cases (50%) veterinary treatment was needed during the whelping; in three cases due to primary incomplete uterine inertia, in one case due to malpositioning of the fetus and in one case due to an oversized pup. The mean age of these bitches was 3.8 (± 1.8) years.

Of 1649 pups that were born in 249 litters, 1254 (76.0%) were subsequently registerered with the Kennel Club. Pup mortality during the period from birth to registration, including stillborn pups was 24.0%: 101 pups (6.1%) were stillborn, 17 pups (1.0%) died in the neonatal period, and 257 pups (15.6%) were euthanised. Information was lacking about what had happened to the remaining 20 pups that were born but never registered. In 155 litters 309 pups were born that were either entirely white or had excessive white markings. A total of 216 of those pups were euthanised because of this disqualifying coat colour (13.0% of all pups born, 84% of all euthanasias).

In the litters in which the number of male pups that were born was reported, 40 out of 401 (9.8%) did not have the testicles in the scrotum at eight weeks of age. Eighty-nine (13.4%) of the 662 male pups that were registered did not have both testicles in the scrotum at eight weeks of age. Mean litter size for litters with some cryptorchid pup was 6.9 (± 2.1) pups born and 5.2 (± 2.0) pups registered. A significant relationship was found between the number of registered male pups per litter and the frequency of cryptorchidism (P = 0.00029), but not between the number of male pups born per litter and this frequency (Table 8). The frequency of cryptorchid male pups was not influenced by the number of female pups in the litter. Looking at the relationship between litter size and the risk for each male pup in a litter to be cryptorchid, a significant positive trend (P = 0.001) was found for male pups in litters with few registered litter mates (of both sexes). No relationship was found between litter size at birth or between the ratio between male and female pups in the litters and the risk for cryptorchidism.

**Table 8:** Frequency distribution of cryptorchid male pups in relation to the number of male pups per litter, A) among all male pups born in litters with known sex distribution at the time of birth (116 litters and 401 male pups) and B) among the registrered pups (253 litters and 662 male pups)

No. of male pups in litter	A. Male pups born		B. Male pups registered	
	
	Cryptorchid male pups (%)	No. of litters	Cryptorchid male pups (%)	No. of litters
0	0	4	0	29
1	7.1	14	20.0	35
2	10.5	19	19.3	57
3	14.8	18	17.5	61
4	9.2	30	9.2	38
5	7.8	18	9.5	21
6	25.0	6	6.1	11
7	0	7	0	1

Umbilical hernias were found in fortyfive pups (3.5% of the registered pups, 2.7% of all the pups born). Eleven pups (0.66% of all the pups born) had a cleft palate and 10 pups (0.78% of the registered pups, 0.60% of all the pups born) had a kinked tail. Eleven pups had other malformations such as a porta-cava shunt, an aorta stenosis, a"hump back" and one was a double fetus.

## Discussion

This study is based on information from breeders answering the questions in the two questionnaires from the Boxer Club. Some additional information was obtained by personal phone calls. The questionnaires were sent out at varying times after the birth of the litters and the answers, therefore, are dependent on the record keeping practices of the breeders. Although most breeders make notes of their breeding activities, some were not able to answer all the questions, and some had misunderstood some of them. In this kind of studies there is also a risk that the outcome of the whelping, in one way or the other, may influence the motivation for the breeder to answer the questions, which may have biased some of the data. The answering frequency of 56.5% is rather high for this kind of studies, but still not high enough to exclude that some of the conclusions drawn may be not entirely representative for the breed. However, the Boxer Club and the breeders participating in this study were highly motivated and the information is, therefore, likely to adequately reflect the degree of the whelping problems in this breed.

In the present study 32% of the individual Boxer bitches needed veterinary treatment during whelping and 28.7% underwent Caesarian section, which is in agreement with the study by Gill (2002) [5] who found that 31.3% of Boxer bitches experienced dystocia, and that Caesarian section was necessary in 25% of the Boxer whelpings. Also, in the Swedish insurance data, the Boxer is described as one of the highrisk breeds for dystocia [8].

It has previously been shown that in the Boston terrier obstructive dystocia is caused by a dorso-ventrally flattened pelvis, and also by some of the pups having a large head [3,4]. The Boxer is also a breed with a fairly large head, but in the bitches in this study the frequency of dystocia was high (27.7%) but the proportion of maternal and fetal dystocia was similar to what has previously been reported for several other breeds [1-3,6], and obstructive dystocia was uncommon. It is, therefore, likely that there is a multifactorial predisposition for dystocia in the Boxer breed and that, like in most other breeds of dogs, primary uterine inertia is the main factor.

In the present study it was found that the need for veterinary assistance during whelping in the Boxer breed increased with age and that the incidence of uterine inertia was significantly higher in bitches that were four years or older than in younger bitches. The risk for other whelping complications than uterine inertia also appeared to be higher in the older bitches. However, no conclusions could be drawn about whether the risk for dystocia was influenced by the parity of the bitch, or whether a bitch experiencing dystocia once may risk dystocia also at the next whelping, because there were too few bitches giving birth to more than one litter during the period of the study, and the questionnaires did not request information on whether the bitches had had previous litters.

Pup death rate in the neonatal period has been reported to be between 10 and 30%, with an average of 12% [7,9,10]. Most of the pups that die succumb during parturition (more than 65%) or the first week after birth [6,7,9]. The comparatively high pup losses between birth and the time of registration seen in the Boxer breed in this study was mainly due to that pups with white coat colour were euthanised. Gill (2002) [5], similarly, found a total pup mortality of 20% in Boxers, with 4.8% stillbirths and 14.3% early neonatal deaths, including the pups euthanised because of too much white in their coat colour. The pup death rate in the present study increased significantly when the parturition lasted for longer than 5 hrs after the birth of the first pup. Gill (2002) [5], studying 500 parturitions in 44 breeds of dogs found that in litters where no mortalities were recorded and no assistance was required during the whelping, the average inter-pup interval was less than 60 min, and that there was a marked increase in perinatal mortality when the inter-pup interval was greater than 90 min. When the inter-pup interval was greater than four hrs, all losses reported were stillbirths.

Gaudet (1985) [2] reported that 39.3% of the bitches with dystocia that were treated with oxytocin, or with calcium in combination with oxytocin, whelped without further treatment, whereas in the study by Darvelid & Linde-Forsberg (1994) [1] the corresponding figure was 30%, and in the present study 25%. This low success rate may be one explanation why many bitches with dystocia are brought to surgery without prior attempts at medical treatment. However, it could also be taken as a sign that more research is needed on both the causes of, and therapy for, uterine inertia in the bitch.

Because the incidence of cryptorchidism in the present study is based on information given by the breeders who usually part with the pups at eight weeks of age, and because the testicles may descend into the scrotum also after this age, the true frequency of cryptorchidism in this material is not known. The Boxer is considered to have a high frequency of cryptorchidism. Hartl (1938) [11] found 23.2% of 168 males to be affected, while Ludwig (1968) [12] found an incidence of 9%. Our findings are contrary to the belief among breeders that cryptorchidism should be more common in litters with many male pups. The inverse relationship found in the present study between the frequency of cryptorchidism and the number of registered male pups in a litter but not for the number of male pups born per litter is difficult to explain. It may, however, be due to that 24% of the male pups that were born in these litters were not registered, and it is not known if their testicles would have been descended into the scrotum at eight weeks of age or not. Additionally, the question about the sex distribution in the litters was only included in the first questionnaire, leading to there being fewer pups in the category "total number of pups born" than in the category "registered pups". Breeders who euthanised white pups (which was the major reason for pups not being registered) most likely did this immediately after birth, and therefore would have had no idea whether the pup would be cryptorchid or not. It is not known whether breeders refrain from registering pups that are cryptorchid at the time of registration of the litter, but this would be contrary to Swedish Kennel Club regulations and no breeder mentioned this circumstance in the questionnaires. Should the breeders have euthanised cryptorchid pups or refrained from registering them when they appeared in litters with many male pups, or if they would be more prone to keep cryptorchid males that were born in litters with few other male pups, this would obviously bias the frequency of cryptorchid males among the registered pups.

## Conclusion

In this study the frequency of dystocia in the Boxer breed was found to be considerably higher than expected in the average dog population. Primary uterine inertia was the most common problem, and increased from four years of age. Some bitches experienced primary uterine inertia during more than one whelping. This is a condition that is considered to have a hereditary component and therefore the bloodlines in which this problem is common should be identified, and breeders should be adviced to avoid breeding from, or combining, such lines. It should, however, be differentiated between bitches that suffer from true primary uterine inertia and those that experience uterine inertia due to carrying only one or two pups, or a very large litter. Also, bitches that have suffered from secondary inertia due to exhaustion from an oversized or malpositioned fetus need not necessarily have a hereditary predisposition for inertia. However, should dystocia due to malpositioned fetuses become overrepresented in some lines the possibility that this is due to some hereditary factor in those lines need to be considered.

## Competing interests

The author(s) declare that they have no competing interests.

## Authors' contributions

The planning of the study was done by CLF, the compilation of data by GP, and the analyses of results and the writing of the manuscript was done in cooperation.

## Supplementary Material

Additional file 1 Appendix 1. Questionnaire to Boxer breeders 1994–1995 Click here for file

Additional file 2 Appendix 2. Questionnaire to Boxer breeders 1996–1997. Whelping survey. Click here for file
