# Supplementary material for: A survey of dystocia in the Boxer breed
Source: Acta Vet Scand. 2007 Mar 21;49(1):8. doi: 10.1186/1751-0147-49-8 (PMC1839103; doi:10.1186/1751-0147-49-8)
Supplement: Additional file 1 — Appendix 1. Questionnaire to Boxer breeders 1994–1995 [file 1751-0147-49-8-S1.doc]

# Appendix 1

Questionnaire to Boxer breeders 1994-1995

FAMILY NAME: FIRST NAME:

ADDRESS:

TOWN:

TELEPHONE: REGIONAL BOXER CLUB:

LITTER

DATE OF BIRTH: DATE:

REGULAR ESTRUS CYCLES: INTERVAL:

EASY TO MATE: PROBLEMS:

FAILED TO GET PREGNANT: VETERINARY EXAMINATION:

HOW LONG TIME AFTER THE BURST OF THE WATER BAG WAS THE FIRST PUP BORN:

DURATION OF WHELPING:

VETERINARY ASSISTANCE: WHY:

C-SECTION: INERTIA: OBSTRUCTED PUP: DEAD FETUS:

CAUSE:

TOTAL No. BORN: LIVE BORN: WHITE COAT: STILLBORN: DIED:

DEAD BY 8 W: REASON:

CULLED BY > 8W: REASON:

TOTAL No REGISTERED:

UMBILICAL HERNIA: CLEFT PALATE: KINKED TAIL: CRYPTORCHID:

OTHER MALFORMATION:

COMMENT AT VETERINARY EXAMINATION:

VETERINARY HELP FOR THE BITCH: REASON:

FOR PUPS: REASON:

MILK SUPPLY: EKLAMPSIA:

DOES THE BITCH VOMIT FOOD FOR THE PUPS: FREQUENCY:

SEPARATION BEFORE 8W: AGE:

ON BREEDING TERMS: BITCH TIRED OF PUPS: OTHER REASON:
