# Supplementary material for: A survey of dystocia in the Boxer breed
Source: Acta Vet Scand. 2007 Mar 21;49(1):8. doi: 10.1186/1751-0147-49-8 (PMC1839103; doi:10.1186/1751-0147-49-8)
Supplement: Additional file 2 — Appendix 2. Questionnaire to Boxer breeders 1996–1997. Whelping survey. [file 1751-0147-49-8-S2.doc]

# Appendix 2

Questionnaire to Boxer breeders 1996-1997

Whelping survey

Breeder:

Litter:

Date of birth:

1. **Was veterinary help needed during the whelping** 🞑 yes 🞑 no if no, continue to question 7.

1. **If veterinary help was needed, was the reason**

🞑 Straining never started

🞑 Straining was weak

🞑 Straining ceased after one/a few of the pups were born. No. of pups delivered ……….

🞑 Strong strainings but no pup was born

🞑 Other reason ………………………….

1. **If x-ray/ultrasound examination was done, was the following found**

🞑 Malposition of fetus

🞑 Only 1 or few pups

🞑 Dead fetus/fetuses

🞑 Many pups/large litter

🞑 Other findings ……………..

1. **What medical treatment or veterinry help was given**

Calcium i.v. 🞑 yes 🞑 no 🢅 🞑 no result

🞑 strainings started …………. number of pups were born

Oxytocin 🞑 yes 🞑 no 🞑 no result

🞑 strainings started …………….number of pups were born

Forceps delivery 🞑 yes 🞑 no ………..number of pups

Caesarian section 🞑 yes 🞑 no

1. **Litter size**

How many pups were there in the litter ……………st

How many were born ”naturally” ………….st

How many were delivered by forceps ………….st

How many were delivered by Caesarian section ………st

How many of those pups were alive …………..st

1. **Veterinarians diagnosis on the claim form to the insurance company**……………………………
2. **For how long time did the whelping last** (from first to last pup)?…………..
3. **Litter information**

Total number of pups born …….. from which.…..... were males and ……... bitches

Number of stillborn pups ………..

Number of white/too much white …………

Number of culled pups ……….. of which ……..were malformed ……… white/too much white

Totalt number of registered pups……..

9. **Heritable defects (give numbers)**

Umbilical hernia………. Cleft palate ……… Kinked tail………..

Cryptorchid ………....... Other malformations……………

**Thank you for taking the time to fill in this form. If you wish to add more details please use the other side.**
